# Protease-Sensitive Conformers in Broad Spectrum of Distinct PrP^Sc^ Structures in Sporadic Creutzfeldt-Jakob Disease Are Indicator of Progression Rate

**DOI:** 10.1371/journal.ppat.1002242

**Published:** 2011-09-08

**Authors:** Chae Kim, Tracy Haldiman, Yvonne Cohen, Wei Chen, Janis Blevins, Man-Sun Sy, Mark Cohen, Jiri G. Safar

**Affiliations:** 1 Department of Pathology, School of Medicine, Case Western Reserve University, Cleveland, Ohio, United States of America; 2 National Prion Disease Pathology Surveillance Center, School of Medicine, Case Western Reserve University, Cleveland, Ohio, United States of America; University of Alberta, Canada

## Abstract

The origin, range, and structure of prions causing the most common human prion disease, sporadic Creutzfeldt-Jakob disease (sCJD), are largely unknown. To investigate the molecular mechanism responsible for the broad phenotypic variability of sCJD, we analyzed the conformational characteristics of protease-sensitive and protease-resistant fractions of the pathogenic prion protein (PrP^Sc^) using novel conformational methods derived from a conformation-dependent immunoassay (CDI). In 46 brains of patients homozygous for polymorphisms in the PRNP gene and exhibiting either Type 1 or Type 2 western blot pattern of the PrP^Sc^, we identified an extensive array of PrP^Sc^ structures that differ in protease sensitivity, display of critical domains, and conformational stability. Surprisingly, in sCJD cases homozygous for methionine or valine at codon 129 of the PRNP gene, the concentration and stability of protease-sensitive conformers of PrP^Sc^ correlated with progression rate of the disease. These data indicate that sCJD brains exhibit a wide spectrum of PrP^Sc^ structural states, and accordingly argue for a broad spectrum of prion strains coding for different phenotypes. The link between disease duration, levels, and stability of protease-sensitive conformers of PrP^Sc^ suggests that these conformers play an important role in the pathogenesis of sCJD.

## Author Summary

Sporadic Creutzfeldt-Jakob disease (sCJD) is the most common human prion disease worldwide. This neurodegenerative disease, which is transmissible and invariably fatal, is characterized by the accumulation of an abnormally folded isoform (PrP^Sc^) of a host-encoded protein (PrP^C^), predominantly in the brain. Most researchers believe that PrP^Sc^ is the infectious agent and five or six subtypes of sCJD have been identified. Whether or not these subtypes represent distinct strains of sCJD prions is debated in the context of the extraordinary variability of sCJD phenotypes, frequent co-occurrence of different PrP^Sc^ fragments in the same brain, and the fact that up to 90% of protease-sensitive PrP^Sc^ eludes the conventional analysis because it is destroyed by protease treatment. Using novel conformational methods, we identified within each clinical and pathological category an array of PrP^Sc^ structures that differ in protease-sensitivity, display of critical domains, and conformational stability. Each of these features offers evidence of a distinct conformation. The link between the rate at which the disease progresses, on the one hand, and the concentration and stability of protease-sensitive conformers of PrP^Sc^ on the other, suggests that these conformers play an important role in how the disease originates and progresses.

## Introduction

Prions cause a group of fatal and rapidly progressing neurodegenerative diseases, originally described as transmissible spongiform encephalopathies (TSEs) [1], [2]. The most common of these diseases is sporadic Creutzfeldt-Jakob disease (sCJD), which accounts for ∼85% of all CJD cases worldwide [3]. Although 40 years ago sCJD was shown to be transmissible to nonhuman primates [4], its pathogenesis remains enigmatic.

Most researchers today believe that all prion diseases are caused by the accumulation of an aberrantly folded isoform, termed PrP^Sc^, of the prion protein PrP [5]. Having a basic amino acid composition and an unstructured N-terminus, PrP can assume at least two conformations: (1) native, α-helix–rich PrP^C^ and (2) disease-causing, β-sheet–rich PrP^Sc^ [6]–[8]. The latter represents a misfolded isoform of the normal cellular prion protein PrP^C^, which is host-encoded by the chromosomal gene PRNP and expressed at different levels in mammalian cells [9]. Yet despite the impressive progress that has been made in understanding the molecular basis of prion diseases, the molecular mechanism of initial misfolding and the high-fidelity replication of the pathogenic conformation of PrP^Sc^ in vivo both remain elusive [2], [10]–[12].

Many lines of evidence from experiments with laboratory prion strains support the view that the phenotype of the disease—its distinctive incubation time, clinical features, and brain pathology—is enciphered in the strain-specific conformation of PrP^Sc^ [13]–[17]. Although remarkable progress has been made in understanding the structure of laboratory strains of rodent prions [2], [10], [18]–[20], knowledge of the molecular basis of human prion diseases has lagged behind. Researchers generally agree that the genotype at codon 129 of the chromosomal gene PRNP underlies susceptibility to these diseases and, to some degree, their phenotype [21]. However, in contrast to the experiments with laboratory rodent prion strains, in which the digestion of brain PrP^Sc^ with proteolytic enzyme proteinase K (PK) consistently results in a single protease-resistant domain with mass ∼19 kDa, the outcome in sCJD is more complex. Distinctive glycosylation patterns and up to four PK-resistant fragments of the pathogenic prion protein (rPrP^Sc^) found in sCJD brains are easily distinguishable on western blot (WB) [14], [21]–[25]. The WB findings together with PRNP gene polymorphism led Parchi, Gambetti, and colleagues to posit a clinicopathological classification of sCJD into five or six subtypes; notably, the WB characteristics of PrP^Sc^ breed true upon transmission to susceptible transgenic mice [14], [21], [22]. An alternative classification of the PrP^Sc^ types and their pairing with CJD phenotypes has been proposed by Collinge and collaborators [23], [24], [26], [27]. This classification differs from the previous one in two major aspects: First, it recognizes three (not two) PrP^Sc^ electrophoretic mobilities; and second, it identifies also PrP^Sc^ isoforms with different ratios of the three PrP glycoforms [26]. Although the disease phenotypes of patients with sCJD are remarkably heterogeneous, 21 kDa fragments of unglycosylated PrP^Sc^ (Type 1) frequently differ from the phenotypes associated with the 19 kDa fragments of unglycosylated PrP^Sc^ (Type 2) [14], [21], [22], [28].

Cumulatively these findings argue that the PrP^Sc^ type represents yet an additional major modifier in human prion diseases; accordingly, WB-based clinicopathologic classifications became an important tool in studies of prion pathogenesis in human brains and in transgenic mice models [14], [26]. Now, inasmuch as two distinct PK cleavage sites in PrP^Sc^ Types 1 and 2 most likely stem from distinct conformations, some investigators contend that PrP^Sc^ Types 1 and 2 code distinct prion strains [14], [23], [28], [29]. However, the heterogeneity of sCJD, along with a growing number of studies including bioassays, all suggest that the range of prions causing sCJD exceeds the number of categories recognized within the current WB-based clinicopathologic schemes [30]–[32]. Additionally, recent findings revealed the co-occurrence of PrP^Sc^ Types 1 and 2 in up to 44% of sCJD cases and thus created a conundrum [33]–[38]. Finally, up to 90% of brain PrP^Sc^ in sCJD eludes WB analysis because it is destroyed by proteinase-K treatment, which is necessary to eliminate PrP^C^. Consequently, the conformation or role of this major protease-sensitive (s) fraction of PrP^Sc^ in the pathogenesis of the disease is a subject of speculation [30], [39], [40].

Aiming to advance our understanding of the molecular pathogenesis of human prion diseases, we used the conformation-dependent immunoassay (CDI) [15], [30], [41] to determine the conformational range and strain-dependent molecular features of sCJD PrP^Sc^ in patients who were homozygous for codon 129 of the PRNP gene. Even relatively minute variations in a soluble protein structure can be determined by measuring conformational stability in a denaturant such as Gdn HCl [42]. Utilizing this concept, we designed a procedure in which PrP^Sc^ is first exposed to denaturant Gdn HCl and then exposed to europium-labeled mAb against the epitopes hidden in the native conformation [15]. As the concentration of Gdn HCl increases, PrPSc dissociates and unfolds from native β-sheet-structured aggregates; and more epitopes become available to antibody binding. These experiments involve insoluble oligomeric forms of PrPSc, and denaturation of this protein is irreversible in vitro; consequently the Gibbs free energy change (ΔG) of PrP^Sc^ cannot be calculated [43]. Therefore we chose instead to use the Gdn HCl value found at the half-maximal denaturation ([GdnHCl]1/2) as a measure of the relative conformational stability of PrPSc. The differences in stability reveal evidence of distinct conformations of PrP^Sc^ [15], [42], [43]. Because CDI is not dependent on protease treatment, it allowed us to address fundamental questions concerning the concentration and conformation of different isoforms of sCJD PrPSc, including protease-sensitive (s) and protease-resistant (r) PrPSc. We found a broad spectrum of structures that are likely responsible for the phenotypic heterogeneity of sCJD and we identified the structural characteristics of PrP^Sc^ that are linked to the duration of the disease.

## Results

### Diagnostic classification of sCJD patients homozygous for PRNP codon 129 and disease duration

**Figure 1:**
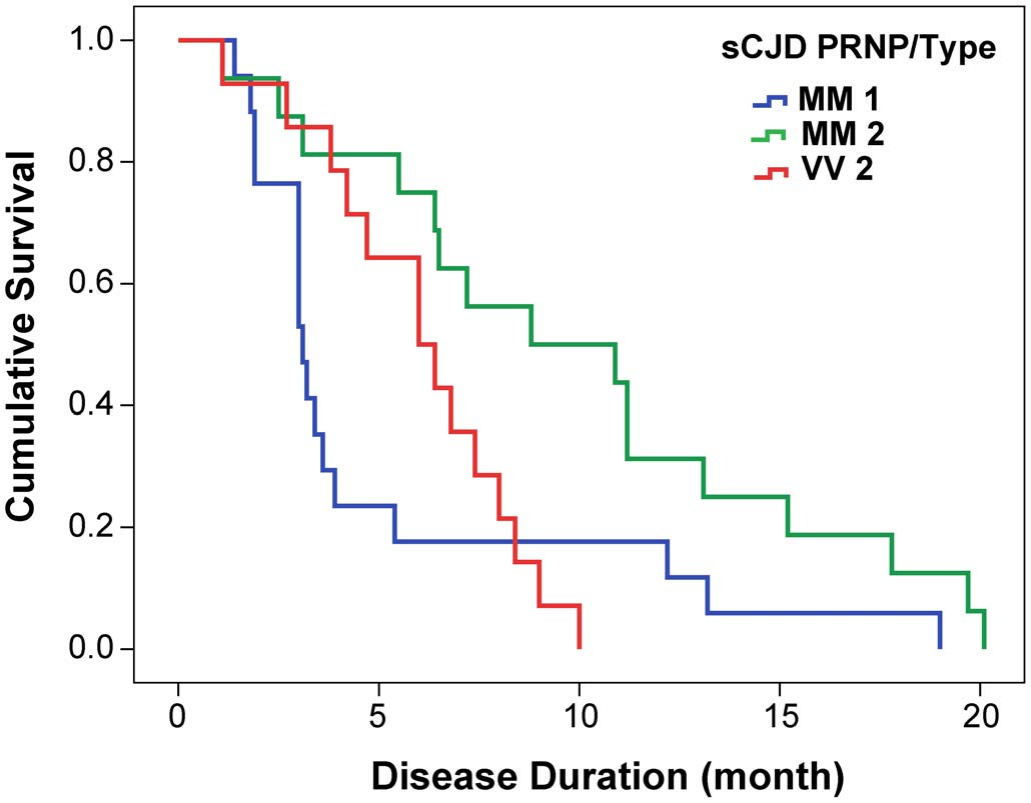
Kaplan-Meier cumulative survival analysis of 46 sCJD cases homozygous for either methionine or valine in codon 129 of PRNP gene and described in this paper. The sCJD cases carrying pure Type 1 PrP^Sc^(129 M) have significantly shorter survival than those with Type 2 PrP^Sc^(129 M) (P = 0.002). The difference in survival of sCJD cases with Type 2 PrP^Sc^(129 V) is significant compared with Type 1 PrP^Sc^(129 M) (P = 0.017) as well as with Type 2 PrP^Sc^(129 M) (P = 0.008).

From 340 patients with an unequivocally definite diagnosis of Type 1 or Type 2 sCJD and who were homozygous for codon 129 polymorphism in the PRNP gene, we selected samples from 46 patients. The descriptive statistics and Kaplan-Meier survival curves indicate that these cases are representative of the whole group collected at NPDPSC and are similar to those previously reported by us and others (Compare **Figure 1** and **Figure S1**, **Table 1**) [21], [38], [44]. As expected, we did not observe statistically significant differences in sex ratio or age at onset of the disease [21], [44]. Kaplan-Meier analyses of survival (**Figure 1**) demonstrated that patients with PrP^Sc^ Type 1 had a significantly shorter disease duration than patients with PrP^Sc^ Type 2 (P = 0.002) despite identical codon 129 MM polymorphism, age, and sex distribution (**Table 1**). Moreover, there is an apparent tendency toward longer survival of patients with Type 2 rPrP^Sc^(129 V) than patients with Type 1 rPrP^Sc^(129 M) (P = 0.017). The difference in survival between patients with Type 2 rPrP^Sc^(129 V) and Type 2 rPrP^Sc^(129 M) was also significant (P = 0.008) with shorter survival of those homozygous for valine (**Figure 1**).

**Table 1:** Demographics of sCJD patients and descriptive statistics of the data.

PRNP Codon 129	MM															VV						
WB Type		1							2							2						
Variable	Units	n	Min	Max	Mean		SEM	Sig	n	Min	Max	Mean		SEM	Sig	n	Min	Max	Mean		SEM	Sig *
**Sex**	F/M	10/6						NS	9/7						NS	7/7						NS
**Age**	years	16	54.0	88.0	68.4	±	2.2	NS	16	53	92	73.9	±	3.1	NS	14	51.0	90.0	71.9	±	2.8	NS
**Disease Duration**	month	16	1.4	13.2	4.2	±	0.9	0.002	16	1.1	20.1	10.0	±	1.5	0.008	14	1.1	10.0	6.0	±	0.7	0.017
**PrP^Sc^**	ng/ml	11	22	547	196	±	53	<0.001	11	28	2996	1203	±	268	<0.001	11	23	1078	207	±	92	NS
**rPrP^Sc^**	ng/ml	16	12	372	118	±	25	<0.001	16	4	1181	422	±	87	0.002	14	3	490	79	±	33	NS
**sPrP^Sc^**	ng/ml	11	11	313	103	±	29	0.002	11	24	1815	691	±	159	0.003	11	3	588	121	±	50	NS
**PrP^Sc^ Gdn HCl_1/2_**	M	11	2.34	3.04	2.77	±	0.07	0.040	11	2.36	3.51	3.04	±	0.10	NS	11	1.34	3.37	2.77	±	0.20	NS
**rPrP^Sc^ Gdn HCl1_/2_**	M	16	2.61	3.34	3.03	±	0.05	<0.001	16	1.57	2.89	2.50	±	0.09	<0.001	14	2.24	3.60	3.21	±	0.09	0.080
**Change in Stability**	ΔFapp	11	0.13	0.72	0.32	±	0.06	<0.001	11	−0.65	0.25	−0.24	±	0.06	0.001	11	−0.32	1.05	0.26	±	0.12	NS

To ensure that the brain homogenate analyzed by CDI contained only Type 1 or 2 rPrP^Sc^, each brain homogenate underwent a second WB (**Figure S2**). The results confirmed the original diagnostic classification but we found two atypical patterns: Case #833 (Type 2 PrP^Sc^(129 M) and Case #162 (Type 2 PrP^Sc^(129 V) revealed, in addition to a band of unglycosylated rPrP^Sc^ with apparent molecular mass ∼19 kDa, a second band with electrophoretic mobility corresponding to mass ∼17 kDa. The observation of different glycoform patterns of PrP^Sc^ in different sCJD cases before protease K treatment and distinct resistance to proteolytic degradation of different glycoforms of PrP^Sc^ is interesting and deserves further investigation.

### Measurement of PrP^Sc^, sPrP^Sc^, and rPrP^Sc^ in sCJD cortex by CDI

To measure the concentration of different forms of PrP^Sc^ in the frontal cortex, we used europium-labeled mAb 3F4 [45] for detection and 8H4 mAb (epitope residues 175–185) [46] to capture human PrP^Sc^ in a sandwich CDI format (**Figure S4**) [30], [47]. The analytical sensitivity and specificity of the optimized CDI for detection of both protease-sensitive (s) and protease-resistant (r) conformers of PrP^Sc^ was previously reported by us and others in numerous publications [15], [30], [41], [48]–[50] and has been shown to be as low as ∼500 fg (∼20 attomoles) of PrP^Sc^. This sensitivity of CDI is similar to the sensitivity of human prion bioassay in Tg(MHu2M)5378/Prnp^0/0^ mice [30].

**Figure 2:**
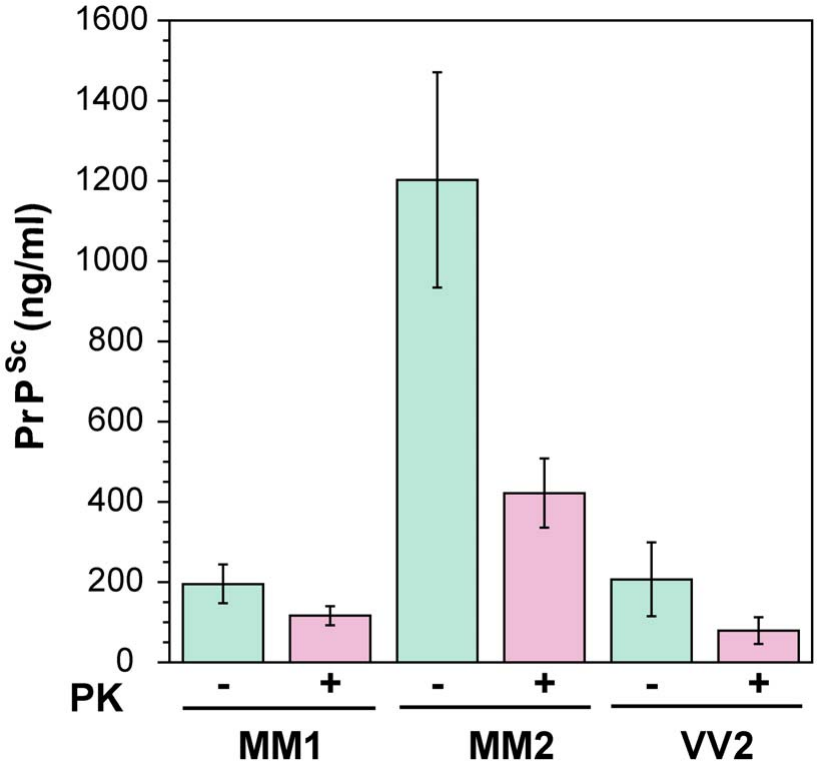
The concentration of PrP^Sc^ and rPrP^Sc^ (PrP 27–30) in 10% homogenate of the frontal cortex of sCJD cases. The PrP^Sc^ was measured by CDI in an aliquot of brain homogenate that was precipitated in the presence of a protease inhibitor cocktail with PTA. The rPrP^Sc^ concentration was determined in a second aliquot treated with PK at concentration equivalent to 3 IU/ml (100 µg/ml) of 10% brain homogenate for one hour at 37°C and precipitated with PTA after blocking PK with the protease inhibitor cocktail. Each sample was measured in triplicate and the concentration was determined by CDI calibrated with recombinant human PrP(23–231) for samples containing full length PrP^Sc^ and with recombinant human PrP(90–231) for samples containing rPrP^Sc^ (PrP 27–30) after PK treatment. The bars are mean ± SEM for each sCJD group.

**Figure 3:**
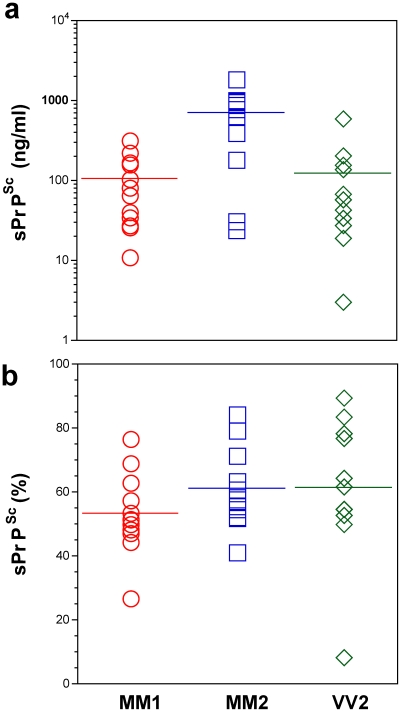
Concentration of sPrP^Sc^ in frontal cortex of sCJD patients. Absolute (a) and relative (b) concentrations of sPrP^Sc^ in frontal cortex of sCJD cases. The higher concentrations of Type 2 PrP^Sc^(129 M) are statistically significant against both Type 1 PrP^Sc^(129 M) (P = 0.002) and Type 2 PrP^Sc^(129 V) (P = 0.003). Each data point represents a unique patient measured by CDI in triplicate and the concentration of sPrP^Sc^ in 10% brain homogenate was calculated from [PrP^Sc^] – [rPrP^Sc^]; the percentage of sPrP^Sc^ is expressed over total PrP^Sc^. The horizontal line represents mean for each group.

First, we determined the concentration of disease-causing PrP^Sc^ in subpopulations of sporadic sCJD patients (**Table 1** **and** **Figure 2**). We observed wide interindividual variations, and approximately sixfold more accumulated PrP^Sc^ in the frontal cortex of patients with Type 2 PrP^Sc^(129 M) than those with Type 1 PrP^Sc^(129 M) or Type 2 PrP^Sc^(129 V). A large portion of PrP^Sc^ in all groups is protease-sensitive, constituting a pool of sPrP^Sc^ conformers (**Table 1** **and** **Figure 3a**). The digestion with proteinase K (PK) was performed with 3 IU/ml (100 µg/ml) of 10% brain homogenate containing 1% sarkosyl for one hour at 37°C. The protocol for PrP^Sc^ digestion, validated in previously published experiments, was selected according to the following criteria: 1) complete digestion of PrP^C^ determined with CDI in control samples; 2) complete shift of the bands of PrP^Sc^ to PrP 27–30 on WBs; 3) unequivocal WB differentiation of Type 1 and Type 2 rPrP^Sc^ in all tested samples [15], [30], [38], [40], [47], [51]. Additionally, the complete digestion of the PrP^Sc^ N-terminus with PK was monitored on WBs in all samples (**Figure S2**).

In patients with Type 2 PrP^Sc^(129 M), significantly higher concentrations of total PrP^Sc^ and sPrP^Sc^ protein (**Table 1**) are associated with extended duration of disease. However, the concentration of sPrP^Sc^ vary greatly between individual patients, with numerous overlapping values between each classification group (**Figure 3a**). Thus, when the concentration of sPrP^Sc^ is expressed as a percentage of total PrP^Sc^, no significant difference between groups appears, and the proportion of sPrP^Sc^ varies from 5% to 90% in individual patients (**Figure 3b**). We concluded from these observations that a major portion of pathogenic sCJD PrP^Sc^ is protease-sensitive and that the highest levels of sPrP^Sc^ are present in Type 2 PrP^Sc^(129 M). The observed large interindividual differences in PK sensitivity likely indicate a broad range of PrP^Sc^ conformers within each PRNP genotype and WB pattern [15], [39]. Since the proteolytic sensitivity of PrP^Sc^ is considered a reliable and constant marker of a distinct prion strain, the data support the conclusion that distinct prion structures are present within each classification group.

### Monitoring the exposure of epitopes 108–112 and 175–185 in native sCJD PrP^Sc^

The partial exposure of epitopes 108–112 and 175–185 in native pathogenic PrP^Sc^ reflects differences in the conformation of native PrP^Sc^ [15], [52]. When we adopted this approach previously, we found considerable differences among eight laboratory prion strains passaged in Syrian hamsters [15]. The denatured state is a reference corresponding to the concentration of PrP^Sc^; the ratio between the fluorescence signal of europium-labeled mAb 3F4 reacting with PrP^Sc^ in the native (N) or completely denatured (D) state represents a relative measure of the degree of exposure of these epitopes.

**Figure 4:**
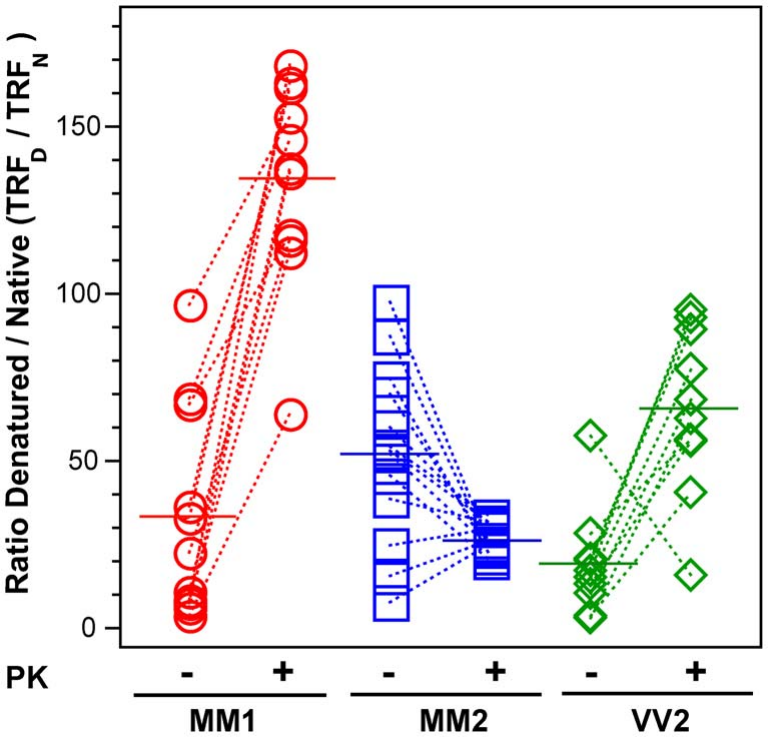
The exposure of 3F4 (108–112) and 8H4 (175–185) epitopes in native PrP^Sc^. The exposure of the epitopes was measured before and after PK treatment in sCJD (red spheres) Type 1 PrP^Sc^(129 M), (blue squares) Type 2 PrP^Sc^(129 M), and (green diamonds) Type 2 PrP^Sc^(129 V). The reactivity of Eu-labeled 3F4 mAb with native (N) and denatured (D) states of proteins was evaluated in a sandwich-formatted CDI and expressed as D/N ratio. The proteins were denatured with 5 M Gdn HCl at room temperature for 30 min. Each data point represents a unique patient measured by CDI in triplicate and the horizontal line corresponds to the mean for the whole group.

The highest D/N PrP^Sc^ ratio was found in patients with Type 2 PrP^Sc^(129 M); and despite a large spread of values, the difference is statistically significant (P = 0.002) (**Figure 4**). PK treatment eliminated most of the exposed 108–112 and 175–185 epitopes in patients with Type 1 PrP^Sc^(129 M) and in patients with Type 2 PrP^Sc^(129 V), resulting in the increased D/N ratios (**Figure 4**). The opposite trend was observed in patients with Type 2 PrP^Sc^(129 M). After PK treatment the PK-induced differences among the three cohorts proved statistically significant to a remarkable degree (P<0.001). Large variations in D/N values exceed what we expect from our experiments with laboratory prion strains [15] and suggest that a high degree of conformational heterogeneity exists in PrP^Sc^ aggregates. Protease treatment change the ratio in all groups and reduced the heterogeneity in MM2 sCJD, and as a result, each group could be reliably differentiated. The increased frequency of exposed epitopes in codon 129 MM samples with Type 2 rPrP^Sc^ after PK treatment is unexpected and may indicate one of three possibilities: that the ligand protecting the 3F4 epitope was removed by PK treatment; that epitope 108–112 was protected by the N-terminus of PrP^Sc^; or that conformational transition resulted in more exposed 108–112 epitopes. Whether the epitopes hindrance in undigested PrPSc is the result of lipid, glycosaminoglycan, nucleic acid, or protein binding to the conformers unique to the MM2 sCJDF PrPSc remains to be established.

### Dissociation and unfolding of sCJD PrP^Sc^, sPrP^Sc^, and rPrP^Sc^ monitored by CDI

First, we asked whether the PTA precipitation has an impact on the stability of PrP^Sc^. This step in the protocol was important for eliminating high concentrations of PrP^C^ and for concentrating PrP^Sc^ in brain samples with relatively low levels of PrP^Sc^. (**Figure S5**). The denaturation curves performed on 5% brain homogenate before PTA precipitation, on PTA pellet and on PTA pellet washed with an excess of H_2_O, were superimposable, an effect which indicated that PTA quantitatively concentrated all PrP^Sc^ conformers and did not influence the stability in CDI. This conclusion accords with numerous previously published data, including bioassays, which indicate that PTA dose not precipitate PrP^C^ and recovers specifically ≥95% of infectious PrP^Sc^ in the pellet, regardless of protease sensitivity or prion strain [15], [30], [53]–[55]. The error of the method does not exceed 5% in monitoring [Gdn HCl]_1/2_ values in the same repeatedly measured brain samples (**Figure S5 and Figure S6**).

Since the dissociation and unfolding of oligomeric PrP^Sc^ may be dependent on protein concentration [42], we first followed the process with CDI at different dilutions of PrP^Sc^ (**Figure S5**). The resulting overlapping dissociation/unfolding curves of PrP^Sc^ with variation in Gdn HCl_1/2_ values <3% indicate that in the 10–250 ng range, the dissociation/unfolding is independent of concentration and is highly reproducible. Furthermore, to ensure the same conditions in all dissociation/unfolding experiments, the PrP^Sc^ content in all samples was maintained at a constant 50 ng/ml concentration. As we observed previously with the western blot technique, the Gdn HCl_1/2_ values obtained with frontal, temporal, parietal, and occipital cortex, thalamus, and cerebellum in three typical sCJD cases were superimposable, indicating that the same conformers of PrP^Sc^ are present in different anatomical areas (data not shown) [38].

**Figure 5:**
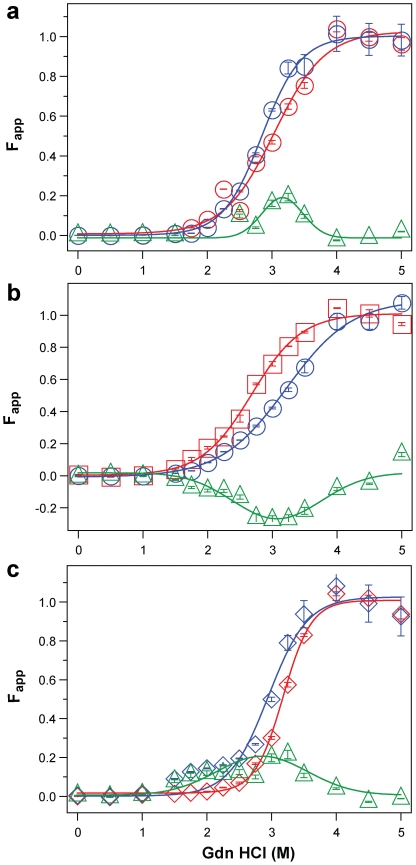
Impact of protease treatment on dissociation and unfolding of PrP^Sc^ monitored with CDI. Typical dissociation and unfolding of (**a**, circles) Type 1 PrP^Sc^(129 M), (**b**, squares) Type 2 PrP^Sc^(129 M), and (**c**, diamonds) Type 2 PrP^Sc^(129 V) followed by CDI before (blue) and after (red) PK treatment; the differences in Fapp values before and after PK treatments are in triangles (green) The curves are the best fit with sigmoidal transition model to determine the midpoint of the curve. The differential values are fitted with Gaussian model and the peak maximum corresponds to the mean stability of sPrP^Sc^. The values of apparent fractional change (Fapp) of each sample aliquot are mean ± SEM obtained from triplicate measurements.

**Figure 6:**
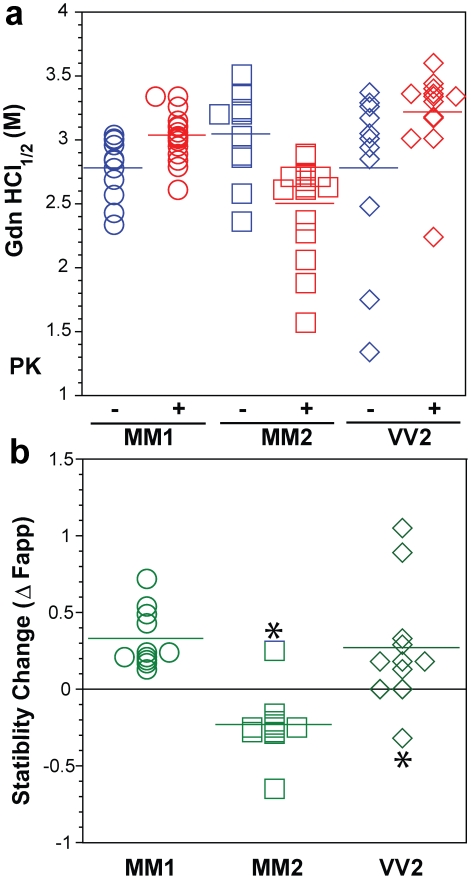
Summary data on conformational stability of PrP^Sc^, rPrP^Sc^, and change in stability induced by PK in 46 sCJD cases. The (**a**) conformational stability of PrP^Sc^ before (blue) and after (red) PK digestion and (**b**) fractional change in stability of PrP^Sc^ induced by PK conformers in individual sCJD samples (circles) Type 1 PrP^Sc^(129 M), (squares) Type 2 PrP^Sc^(129 M), and (diamonds) Type 2 PrP^Sc^(129 V). The stability was determined by CDI and expressed as Gdn HCl_1/2_ or stability change (Δ Fapp) induced by PK. Each symbol represents an individual patient measured in triplicate and the mean level in each group is indicated by the horizontal line.

Next we examined the frontal cortex of individual sCJD patients homozygous for methionine or valine at codon 129 of the PRNP gene. Typical examples of dissociation/unfolding curves are shown in **Figures 5a, 5b, and 5c**. Comparing all sCJD cases, we found a broad range of Gdn HCl_1/2_ values ranging from 1.3 to 3.5 M (**Figure 6a**). Because of the wide spread of values, the difference between the cases with Type 1 and 2 PrP^Sc^(129 M) is only marginally significant (P = 0.040) and there is no statistically significant difference among other groups. The possible cluster of Gdn HCl_1/2_ values at ∼3.0 M is discernible in cases with Type 1 PrP^Sc^(129 M) (**Figure 6a**). We concluded from these experiments that PrP^Sc^ proteins in different brains of sCJD patients display a vast range of unique conformations within each classification group.

### The conformational impact of PK treatment

We next investigated the conformational impact of the proteolytic digestion of sPrP^Sc^ conformers and the loss of N-terminal residues in rPrP^Sc^. The proteolysis of PrP^Sc^ with PK resulted in increased conformational stability in Type 1 rPrP^Sc^(129 M) and Type 2 rPrP^Sc^(129 V) but did not significantly reduce the range of values (**Figure 6a**). In contrast, PK treatment uniformly decreased Gdn HCl_1/2_ values in Type 2 rPrP^Sc^(129 M) (**Figure 6a**). The marked drop in this group's stability is statistically significant to a high degree (P<0.001). Additionally, there is a discernible cluster of Type 2 PrP^Sc^(129 M) cases at ∼2.6 M (**Figure 6a**). We interpret the data as providing evidence of a wide range of unique conformations in each subgroup. Proteolytic treatment selects the conformers having a more stable core in Type 1 rPrP^Sc^(129 M) and Type 2PrP^Sc^(129 V). The opposite effect of PK, as well as decreased stability, was observed in samples with Type 2 PrP^Sc^(129 M). These data suggest that PK treatment generates a unique set of conformers in Type 2PrP^Sc^(129 M), characterized by increased exposure of 108–112 and 175–185 epitopes (**Figure 5**) and, upon PK treatment, decreased stability of the core rPrP^Sc^(129 M).

To investigate the conformational stability of sPrP^Sc^ separately from rPrP^Sc^, we subtracted the relative fractional change in stability of rPrP^Sc^ after PK treatment from the PrP^Sc^ values obtained before PK (**Figures 5a, 5b, and 5c**). The resulting differential curves exhibit Gaussian distribution with the peak at the median stability of sPrP^Sc^; the height and integrated peak area is proportional to the relative fraction of PK-digested conformers. Overall stability of Type 1 sPrP^Sc^ is, as expected, lower than that of rPrP^Sc^ and we estimate, from these data alone, that sPrP^Sc^ conformers constitute 13–72% of the PrP^Sc^ (**Figure 6a**). A larger spread of positive values obtained with Type 2 sPrP^Sc^(129 V) coincides with a generally larger spread of Gdn HCl_1/2_ values in this group. In contrast, the negative differential curves for Type 2 sPrP^Sc^(129 M) indicate that sPrP^Sc^ is more stable than rPrP^Sc^ in this patient group (**Figure 6b**). Notably, the only positive value in this group came from a sample having an atypical 19 and 17 kDa doublet of unglycosylated rPrP^Sc^ on WBs (**Figure 6b** **and Figure S2**). Since the stability of sPrP^Sc^ and of rPrP^Sc^ reflect different initial conformation, the observed spread of values suggests a broad range of unique PrP^Sc^ conformers within each PRNP genotype and WB pattern [**15]**, [39], [56****].

To determine whether unifying trends exist, we examined which PrP^Sc^ characteristics have an impact on duration of the disease in individual patients in all groups using regression analysis. In contrast to analysis of variance (Anova) used to compare MM1, MM2, and VV2 groups (**Table 1**), the regression analysis is testing the relationship between a dependent variable (duration of the disease) and independent variables (e.g., sPrPSc levels) in individual patients. From concentrations of PrP^Sc^, sPrP^Sc^, and rPrP^Sc^, only the levels of sPrP^Sc^ (**Figure S7a**) correlated significantly with longer duration of the disease. The overall dependency is driven mainly by the higher levels of sPrP^Sc^ in Type 2 sPrP^Sc^(129 M) and longer duration of the disease in this subgroup (**Table 1**). Additionally, the measurement of absolute concentration of sPrP^Sc^ is clearly a better indicator of this relationship than the estimate of the relative fraction (percentage) of sPrP^Sc^ (**Figure 3b**). Despite a wide spread of values, this observation corroborates the conclusion, drawn from previous experiments with eight laboratory strains of prion, that incubation time, and by extension duration of the disease, is linked to the higher levels of sPrP^Sc^ [15].

**Figure 7:**
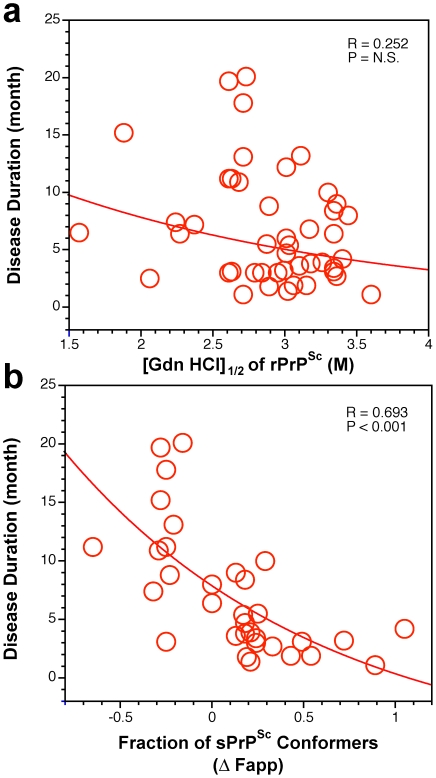
Duration of sCJD correlate with conformational stability of sPrP^Sc^. The relationship between duration of the disease and conformational stability of (**a**) rPrP^Sc^ and (**b**) fraction of sPrP^Sc^ conformers in 46 sCJD patients was analyzed by the regression analysis.

We then analyzed the conformational characteristics of PrP^Sc^. The stability of rPrP^Sc^ clearly did not correlate with duration of the disease in individual cases (**Figure 7a**). In contrast, the change in the stability of PrP^Sc^ upon PK treatment (**Figure S7b**) or relative levels of sPrP^Sc^ conformers eliminated by PK (**Figure 7b**) expressed as a fraction of all conformers, both demonstrated better correlation with duration of the disease than did any other parameter in both Type 1 and Type 2 cases. In contrast to simple measurement of sPrP^Sc^ concentration, the stability assay performed before and after PK treatment cumulatively determines the shift in the stability of PrP^Sc^, change in the slope of the denaturation curve (dissociation/unfolding rate), and relative levels of the sPrP^Sc^ conformers in the total PrP^Sc^ pool. This effect leads to the clear separation of Type 1 from Type 2 sPrP^Sc^(129 M) cases (**Figure 7b**). We interpret these findings as evidence of the differential impact of protease treatment on different conformers, resulting in either increased or decreased stability of the remaining rPrP^Sc^ core (PrP 27–30). Taken together, higher levels of more stable sPrP^Sc^ conformers are associated with extended duration of the disease. Conversely, lower concentrations of unstable sPrP^Sc^ correlate with faster progression of the disease.

## Discussion

The discovery of heritable polymorphic PK cleavage sites and glycosylation patterns in PrP^Sc^ have been used for the initial diagnostic classifications of sCJD cases. In concert with the codon 129 PRNP haplotype, the different rPrP^Sc^ types broadly correlate with distinct disease phenotypes [14], [21], [27]–[29], [57]. The majority of sCJD patients are homozygous for methionine at codon 129 of the PRNP gene; they also accumulate Type 1 rPrP^Sc^ and present with so-called classic sCJD, characterized by rapidly progressive dementia, early myoclonus, visual disturbances including cortical blindness, disease duration of approximately 4 months, and fine punctate (synaptic) deposits of PrP^Sc^ [21], [30]. In contrast, patients with the second most frequent phenotype are homozygous for valine at codon 129 of the PRNP gene, accumulate Type 2 PrP^Sc^ and manifest a different disease course, with early ataxia, predominant extra-pyramidal symptoms, relatively late-onset dementia in the extended course of the disease, and large plaque-like deposits of PrP^Sc^ [21].

In the increasing number of subsequent sCJD cases which were examined with more sensitive and specific techniques, investigators began to recognize the extensive variability of the sCJD phenotypes, as well as the extreme complexity of brain immunohistochemistry and western blot patterns of PrP^Sc^ [25], [32], [37], [38], [57]–[59]. Although the western blot systems provided early evidence that molecular characteristics of PrP^Sc^ are transmissible, evidence regarding the original conformation of PrP^Sc^ remains indirect and limited to the most protease-resistant fractions. Because variable fractions of PrP^Sc^ are protease-sensitive, we decided to determine the conformational characteristics directly, by using CDI. This method allowed us to compare the conformational features of human PrP^Sc^ independently of proteolytic treatment and in addition provided quantitative data on levels of PrP^Sc^, sPrP^Sc^, and rPrP^Sc^ [15], [30]. The CDI techniques represent a major improvement over previously used semi-quantitative WB-based methods, the finding that has been independently confirmed by another group [60], [61]. The dissociation and unfolding of PrPSc in a presence of increasing concentration of Gdn HCl can be described as follows: [PrP^Sc^]_n_→[sPrP^Sc^]_n_→iPrP→uPrP, where [PrP^Sc^]_n_ are native aggregates of PrP^Sc^, [sPrP^Sc^]_n_ are soluble protease-sensitive oligomers of PrP^Sc^, iPrP is an intermedite, and uPrP is completely unfolded (denatured) PrP [7], [43], [62]. The CDI monitors the global transition from native aggregates to fully denatured monomers of PrPSc. In contrast, the WB based techniques monitor either the partial solubilization of PrP^Sc^ [63] or conversion of rPrPSc to protease-sensitive conformers [16] after exposure to denaturant. As a result, the stability data on soluble protease sensitive oligomers and intermediates of PrPs cannot be obtained with WB techniques and lead to the markedly underestimated values [60].

### Levels and role of PrP^Sc^ isoforms in the pathogenesis of sCJD

The sixfold difference in concentrations of PrP^Sc^ between Type 1 and Type 2 PrP^Sc^(129 M) (**Figure 2**) revealed in the frontal cortex by means of CDI was surprising, even though some variability was to be expected due to differences in the predominantly affected areas in distinct sCJD phenotypes [30]. The average levels of PrP^Sc^ are up to 100-fold lower than those in standard laboratory prion models such as Syrian hamsters infected with Sc237 prions [15]; and together with the up to 100-fold variability within each phenotypic group, these lower levels of PrP^Sc^ may partially explain why some sCJD cases are difficult to transmit, and why lower endpoint titers are obtained with human prions in transgenic mice expressing human PrP^C^ [14], [26], [30], [64].

As we observed previously, up to 90% of the pathogenic prion protein was protease-sensitive [30]. In this study, we found the highest concentrations in Type 2 PrP^Sc^(129 M). The broad range of absolute and relative levels of rPrP^Sc^ and sPrP^Sc^ offers evidence of a broad spectrum of PrP^Sc^ molecules differing in protease sensitivity in each group with an identical polymorphism at codon 129 of the PRNP gene and an identical WB pattern (**Figure 3**). Moreover, these findings signal the existence of a variety of sCJD PrP^Sc^ conformers; and since protease sensitivity is one of the characteristics of prion strains, they also suggest that distinct sCJD prion strains exist [15], [30], [31], [58], [62], [65].

### Structural heterogeneity and origin of sCJD PrP^Sc^

The CJD cases studied in this paper represent 75–90% of all clinical and pathologic diagnostic categories of sCJD [21]. In order to allow unequivocal interpretation of the CDI data, we had to exclude sCJD patients heterozygous for codon 129 polymorphism in the PRNP gene, even though they represent ∼15–20% of sCJD cases. The CDI cannot differentiate PrP^Sc^ with codon 129 M from V in a mixture which is present in sCJD heterozygots, and therefore we were unable to differentiate the conformational impact of codon 129 polymorphism. We also excluded the VV1 type of sCJD because of its rarity. This rare form of sCJD constitutes ∼1% of all sCJD cases and we did not collect enough cases to allow statistical comparison with the other groups [21].

The heterogeneity of PrP^Sc^ conformations found with CDI within sCJD patients homozygous for codon 129 plymorphism of the PRNP gene is remarkable (**Table 1** **and** **Figure 6**), with a range corresponding to that of stabilities found in more than ∼30 distinct strains of de-novo and natural laboratory rodent prions studied up to now [15], [16], [66]. The high sensitivity and reproducibility of CDI, together with broad inter-individual variability detected with techniques based on three different principles—PK sensitivity, epitope exposure, and conformational stability—all indicate that the intragroup variations did not originate in the CDI technique but rather reflect differences in the structure of PrP^Sc^ in different patients. The intriguing effect of PK treatment on the stability of Type 2 PrP^Sc^(129 M) suggests that the protease-resistant core of Type 2 was profoundly destabilized. Since sCJD cases with Type 2 PrP^Sc^(129 M) have remarkably extended disease durations, the molecular mechanism underlying this effect calls for detailed investigation.

Several theories have been proposed to explain the origin of sCJD. One argues for spontaneous somatic mutations in PRNP; another, for rare stochastic conformational changes in PrP^C^ [26], [67]. Yet a third hypothesis holds that low levels of PrP^Sc^ are normally present and cleared, but rise to pathogenic levels when the clearance mechanism fails [40]. Cumulatively, our findings indicate that sCJD PrP^Sc^ exhibit extensive conformational heterogeneity. Whether this heterogeneity originates in a stochastic misfolding process that generates many distinct self-replicating conformations [26], [67] or in a complex process of evolutionary selection during development of the disease [17] remains to be established.

### Protease-sensitive conformers of PrP^Sc^

We discovered this fraction of PrP^Sc^ while developing a conformation-dependent immunoassay (CDI), which does not require proteolytic degradation of ubiquitous PrP^C^ [15]. Although the original definition of sPrP^Sc^ was only operational, considerable additional data demonstrate that (1) sPrP^Sc^ replicates in vivo and in vitro as an invariant and major fraction of PrP^Sc^; (2) sPrP^Sc^ separates from rPrP^Sc^ in high speed centrifugation; and (3) the proteolytic sensitivity of PrP^Sc^ can reliably differentiate various prion strains [15], [30], [31], [58], [62], [65]. Accumulation of sPrP^Sc^ precedes protease-resistant product (rPrP^Sc^) in prion infection [40], [68]; and up to 90% of PrP^Sc^ accumulating in CJD brains consists of sPrP^Sc^ [30]. Thus, the detection by CDI of sPrP^Sc^ as a disease-specific marker is widely regarded as a more reliable basis for diagnosing prion diseases. This improved detection led to the discovery of a new human prion disorder, variably protease-sensitive prionopathy (VPSPr) [15], [30], [39], [69], [70]. It is noteworthy that protease-sensitive synthetic prions generated in vitro during polymerization of recombinant mouse PrP into amyloid fibers produced upon inoculation into wild mice prions composed exclusively of sPrP^Sc^ [66].

In laboratory rodent prion models, we found that levels of sPrP^Sc^ varied with the incubation time of the disease [15] but the molecular mechanism of this link was unknown [15], [30], [40]. Subsequent experiments with yeast prions indicated that replication rate may be an inverse function of the stability of misfolded protein [71]. The hypothesis based on these experiments posits that the less stable prions replicate faster by exposing more available sites for growth of the aggregates. Additionally, experiments with laboratory and synthetic prions in mouse suggested that the yeast prion principle may apply to mammalian prions as well. However, these experiments were based entirely on the correlation of the shorter incubation time of mouse inoculated with PrP^Sc^ that on WBs converted to protease-sensitive isoforms at a lower denaturant concentration, whereas the replication rates of mammalian prions were never determined [72].

In this paper we determined the conformational features and stability of human sPrP^Sc^ in sCJD. The data indicate that the levels as well as stability are linked to the progression rate of the disease. Despite the inevitable influence of variable genetic background and the potential difficulties in evaluating initial symptoms, the disease progression rate and incubation time jointly represent an important parameter, which is influenced by replication rate, propagation, and clearance of prions from the brain [2], [40]. The correlations among the levels of sPrP^Sc^, the stability of sPrP^Sc^, and the duration of the disease found in this study all indicate that sPrP^Sc^ conformers play an important role in the pathogenesis. When sPrP^Sc^ is less stable than rPrP^Sc^, the difference in stability correlates with less accumulated sPrP^Sc^ and shorter duration of the disease. Conversely, when sPrP conformers are more stable than rPrP^Sc^, we observe the opposite effect—more accumulated sPrP^Sc^ and extended disease duration. It remains to be determined if these effects represent an outcome of different replication rates and clearance, or whether they stem from as yet unknown aspects of the pathogenesis of sCJD.

## Materials and Methods

### Ethics statement

All procedures were performed under protocols approved by the Institutional Review Board at Case Western Reserve University. In all cases, written informed consent for research was obtained from patient or legal guardian and the material used had appropriate ethical approval for use in this project. All patient's data and samples were coded and handled according to NIH guidelines to protect patients' identities.

### Patients and clinical evaluations

We selected 46 representative subjects from a group of 340 patients with definitive diagnosis of sCJD. The criteria for inclusion were (1) availability of clinical diagnosis of CJD according to WHO criteria [73]–[75] and clearly determined and dated initial symptoms upon neurological examination to ascertain the disease duration; (2) methionine or valine homozygous at codon 129 of the human prion protein (PrP) gene (PRNP); (3) unequivocal classification as pure Type 1 or Type 2 sCJD according to WB pattern; (4) unequivocal classification of pathology as definite Type 1 or 2 at the National Prion Disease Pathology Surveillance Center (NPDPSC) in Cleveland, OH; (5) demographic data distribution within 95% confidence interval of the whole group resulting in no difference between selected cases and the whole group in any of the statistically followed parameters.

Retrospective charts review was carried out for all subjects, with particular attention to the documented initial cardinal clinical signs of sCJD such as cognitive impairment, ataxia, and myoclonus [73]–[75]. We also reviewed the findings on electroencephalography, brain magnetic resonance imaging, and CSF markers when available.

### Brain samples and PRNP gene sequencing

All Type 1–2 patients or uncertain cases were excluded from this study. DNA was extracted from frozen brain tissues in all cases, and genotypic analysis of the PRNP coding region was performed as described [29], [30], [76]. On the basis of diagnostic pathology, immunohistochemisty, and western blot (WB) examination of 2 or 3 brain regions (including frontal, occipital and cerebellum cortices) with mAb 3F4, the pathogenic PrP^Sc^ was classified as (1) Type 1 PrP^Sc^(129 M) (n = 16); (2) Type 2 PrP^Sc^ (129 M, n = 16); or (3) Type 2 PrP^Sc^ (129 V, n = 14). Patients lacked pathogenic mutations in the PRNP and had no history of familial diseases or known exposure to prion agents. These cases underwent additional detailed WB analyses of the PrP^Sc^ so that we could ascertain the accuracy of their original classification and confirm that the same brain homogenate analyzed by CDI contained pure Type 1 PrP^Sc^(129 M), Type 2 PrP^Sc^(129 M), and Type 2 PrP^Sc^(129 V).

Coronal sections of human brain tissues were obtained at autopsy and stored at 80°C. Three 200–350 mg cuts of frontal (superior and more posterior middle gyri) cortex were taken from each brain and used for molecular analyses. The other symmetric cerebral hemisphere was fixed in formalin and used for histologic and immunohistochemical purposes.

### Brain homogenates and precipitation of prions with PTA

Slices of tissues weighing 200–350 mg were first homogenized to a final 15% (w/v) concentration in calcium- and magnesium-free PBS, pH 7.4, by 3 75 s cycles with Mini-beadbeater 16 Cell Disrupter (Biospec, Bartlesville, OK). The homogenates were then diluted to a final 5% (w/v) in 1% (v/v) sarkosyl in PBS, pH 7.4 and rehomogenized. After clarification at 500× g for 5 min., one aliquot of the supernatant was treated with protease inhibitors (0.5 mM PMSF and aprotinin and leupeptin at 5 ug/ml, respectively). The second aliquot was treated with 50 µg/ml of proteinase K (Amresco, Solon, OH) for 1 h at 37°C shaking 600 rpm on Eppendorf Thermomixer (Eppendorf, Hauppauge, NY) and PK was blocked with PMSF and aprotinin-leupeptin cocktail. Both aliquots were precipitated with final 0.32% (v/v) NaPTA after 1 h incubation at 37°C as described [15]. The samples were spun 30 min at 14,000× g in Allegra X-22R tabletop centrifuge (Beckman Coulter, Brea, CA) and the pellets were resuspended in 250 ul of deionized water containing protease inhibitors (0.05 mM PMSF, aprotinin and leupeptin at 1 ug/ml each, respectively, and stored for analysis at −80°C.

### Western blots

Both PK-treated and untreated samples were diluted 9-fold in 1× Laemmli Buffer (Bio-Rad, Hercules, CA) containing 5% (v/v) beta-mercaptoethanol (ME) and final 115 mM Tris-HCl, pH 6.8. Samples were heated for 5 min at 100°C and ∼2 ng of PrP per lane was loaded onto 1 mm 15% Polyacrylamide Tris-HCl, SDS-PAGE gels (Bio-Rad) mounted in Bio-Rad Western Blot apparatus. After electro-transfer to Immobilon-P Transfer Membranes (Millipore, Bedford, MA), the membranes were blocked with 2% (w/v) BSA in TBS containing 0.1% of Tween 20 (v/v) and 0.05% (v/v) Kathon CG/ICP (Sigma, St. Louis, MO). The PVDF membranes were developed with 0.05 ug/ml of biotinylated mAb 3F4 (Covance, Princeton, NJ) followed by 0.0175 ug/ml Streptavidin-Peroxidase conjugate (Fisher Scientific, Pittsburg, PA) or with ascitic fluid containing mAb 3F4 (kindly supplied by Richard Kascsak) diluted 1∶20,000 followed by Peroxidase-labeled sheep anti-mouse IgG Ab (Amersham, Piscataway, NJ) and diluted 1∶3000. The membranes were developed with the ECL Plus detection system (Amersham) and exposed to Kodak BioMax MR Films (Fisher Scientific) or Kodak BioMax XAR Films (Fisher Scientific).

### Conformation-dependent immunoassay (CDI)

The CDI for human PrP was performed as described previously [30], [47], with several modifications. First, we used white Lumitrac 600 High Binding Plates (E&K Scientific, Santa Clara, CA) coated with mAb 8H4 (epitope 175–185) [46] in 200 mM NaH_2_PO_4_ containing 0.03% (w/v) NaN_3_, pH 7.5. Second, aliquots of 20 µl from each fraction containing 0.007% (v/v) of Patent Blue V (Sigma) were directly loaded into wells of white strip plates prefilled with 200 µl of Assay Buffer (Perkin Elmer, Waltham, MA). Finally, the captured PrP was detected by a europium-conjugated [15] anti-PrP mAb 3F4 (epitope 108–112) [45] and the time-resolved fluorescence (TRF) signals were measured by the multi-mode microplate reader PHERAstar Plus (BMG LabTech, Durham, NC). The recHuPrP(90–231,129 M) and PrP(23–231,129 M) used as a calibrant in the CDI was a gift from Witold Surewicz, and preparation and purification have been described previously [77]. The initial concentration of recombinant human PrP(23–231) and PreP(90–231) was calculated from absorbance at 280 nm and molar extinction coefficient 56650 M^−1^ cm^−1^ and 21640 M^−1^ cm^−1^, respectively. The purified recombinant proteins were dissolved in 4 M GdnHCl and 50% Stabilcoat (SurModics, Eden Prairie, MN), and stored at −80°C. The concentration of PrP was calculated from the CDI signal of denatured samples using calibration cure prepared with either recPrP(23–231) for samples containing full length PrP^Sc^ or recPrP(90–231) for samples containing truncated rPrP^Sc^ (PrP 27–30) after proteinase-K treatment. This separate calibration was necessary due to the ∼3.5-fold lower affinity of mAb 3F4 with full length hurman PrP(23–231,129 M) compared to PrP(90–231,129 M) (**Figure S3**).

### Monitoring dissociation and unfolding of PrP^Sc^ by CDI

The denaturation of human PrP^Sc^ was performed as described previously [15], with several modifications. Frozen aliquots of PrP^Sc^ were thawed, sonicated 3×5 s at 60% power with Sonicator 4000 (Qsonica, Newtown, CT), and the concentration was adjusted to constant ∼50 ng/ml of PrP^Sc^. The 15 µl aliquots in 15 tubes were treated with increasing concentrations of 8 M GdnHCl containing 0.007% (v/v) Patent Blue V (Sigma, St. Louis, MO) in 0.25 M or 0.5 M increments. After 30 min incubation at room temperature, individual samples were rapidly diluted with Assay Buffer (Perkin Elmer, Waltham, MA) containing diminishing concentrations of 8 M GdnHCl, so that the final concentration in all samples was 0.411 M. Each individual aliquot was immediately loaded in triplicate to dry white Lumitrac 600, High Binding Plates (E&K Scientific, Santa Clara, CA), coated with mAb 8H4, and developed in accordance with CDI protocol using europium-labeled mAb 3F4 for detection [15], [30], [41], [78].

The raw TRF signal was converted into the apparent fractional change of unfolding (Fapp) as follows: F = (TRF_OBS_−TRF_N_)/(TRF_U_−TRF_N_) where TRF_OBS_ is the observed TRF value, and TRF_N_ and TRF_U_ are the TRF values for native and unfolded forms, respectively, at the given Gdn HCl concentration [7]. To determine the concentration of Gdn HCl where 50% of PrP^Sc^ is unfolded ([Gdn HCl]_1/2_), the data were fitted by least square method with a sigmoideal transition model (Equation 1):
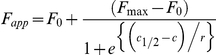



The apparent fractional change (F) in the TRF signal is the function of Gdn HCl concentration(c); c_1/2_ is the concentration of Gdn HCl at which 50% of PrP^Sc^ is dissociated/unfolded and r is the slope constant. To determine the impact of protease treatment on the conformational stability of PrP^Sc^, the values of fractional change after PK were subtracted from F_app_ values obtained before PK (ΔF_app_ = F^0^−F^PK^) and then fitted with a Gaussian model to estimate the proportion and average stability of sPrP^Sc^ conformers (Equation 2):
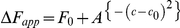
In this model, the Pk-induced fractional change is ΔF, F_0_ is fractional change at 0 concentration of Gdn HCl, and c_0_ is the Gdn HCl concentration at the maximum height A of the peak.

### Statistical analysis

We investigated the effect of the following demographic and laboratory variables on survival: sex; age at onset; duration of the disease; electrophoretic Type of PrP 27–30; and the concentration and stability of PrP^Sc^ in Gdn HCl before and after PK treatment [15]. Cumulative survival curves were constructed by the Kaplan–Meier method, both overall and by stratifying for each of the above variables. For each type of PrP^Sc^ and PRNP gene polymorphism, we report descriptive statistics and the overall survival times stratified for each variable. In the comparison of different patient groups, *P* values were calculated using Anova. Comparisons of survival curves among groups were carried out by the log rank (Mantel-Cox) and generalized Wilcoxon test. To evaluate the dependency of disease duration upon the concentration and stability of PrP^Sc^ in individual CJD cases, the data were analyzed by non-linear regression using the logistic function or the nonlinear models with the best fit. To obtain significance and to compare the relative importance of each characteristic of PrPSc, we used ANOVA and F statistics with regression mean square (MSR) divided by the residual mean square (MSE). All the statistical analyses were performed using SPSS 17 software (SPSS Inc., Chicago, IL).

## Supporting Information

Figure S1 Kaplan-Meier cumulative survival analysis of 340 sCJD cases homozygous for either methionine (n = 288) or valine (n = 52) in codon 129 of PRNP gene from which were selected the 46 cases described in this paper. The sCJD cases carrying pure type 1 PrP^Sc^(129 M) (n = 266) have significantly shorter disease duration than those with type 2 PrPSc(129 M) (n = 22, P<0.001). The intermediate duration of the disease observed in sCJD cases with type 2 PrP^Sc^(129 V) (n = 52) is significant compared with type 1 PrP^Sc^(129 M) (P<0.001) or type 2 PrP^Sc^(129 M) (P<0.001).
(TIF)

Figure S2 Typical WB analysis of PrP^Sc^ and rPrP^Sc^ in sCJD cases. PrP^Sc^ from 5% brain homogenate in PBS, pH 7.4, containing 1% Sarcosyl was precipitated with PTA either before (left lanes) or after (right lanes) digestion with 50 µg/ml of PK at 37°C for 1 h. Note the 19 and 17 kD doublets of unglycosylated bands of PrP^Sc^ in MM2 Case #7-927 and VV2 Case #8-848. The rPrP^Sc^ bands in Case VV2 9-434 became visible only after prolonged exposure (data not shown). Internal controls of type 1 (T1) or type 2 (T2) rPrP^Sc^(129 M) were incorporated in each WB.
(TIF)

Figure S3 Calibration of CDI with (**squares**) full length (PrP23–231, 129 M) or (**circles**) truncated (PrP90–231, 129 M) prion protein. The truncated (PrP90–231, 129 M) prion protein corresponds to the human brain PrP 27–30 after proteinase K treatment. Time-resolved fluorescence (TRF) is reported in counts per minute (cpm) from triplicate measurement ± SEM. The initial concentrations of recombinant human PrP(23–231) and PreP(90–231) were calculated from absorbance at 280 nm and molar extinction coefficient 56650 M^−1^ cm^−1^ and 21640 M^−1^ cm^−1^, respectively.
(TIF)

Figure S4 The (**a**) raw time-resolved fluorescence (TRF) data and (**b**) end-point sensitivity in detection of sCJD PrPSc with CDI before and after proteinase K treatment in different cases of sCJD and a case of other neurological disorder (OND). To obtain values for total PrPSc, CDI was performed in an aliquot of brain homogenate that was precipitated in the presence of a protease inhibitor cocktail with PTA. To obtain CDI readings for rPrPSc, samples were treated with PK at concentration equivalent to 3 IU/ml (100 µg/ml) of 10% brain homogenate for one hour at 37°C and precipitated with PTA after blocking PK with the protease inhibitor cocktail. The 8H4 mAb was used {Zanusso, 1998 #4838} for capture and Eu-labeled 3F4 mAb for detection under native (N) and denatured (D) conditions {Safar, 2005 #6826;Safar, 2002 #5989;Safar, 1998 #4776}. The (D – N) values of time-resolved fluorescence (TRF) measured in counts per minute (cpm) are directly proportional to the concentration of PrPSc [Safar, 2005 #6826;Safar, 2002 #5989;Safar, 1998 #4776]. Data points and bars represent average ± standard deviation (SD) obtained from three or four independent measurements.
(TIF)

Figure S5 The dissociation and unfolding of PrP^Sc^(129 M) monitored by CDI in 5% brain homogenate (**circles**), in PTA pellet (**squares**), and washed PTA pellet (**triangles**). The brain homogenate and PTA precipitation was performed as described in the Method section. For wash, the PTA pellet was resuspended in 1 ml of H_2_O containing protease inhibitors, spun at 14,000 G for 30 min, and then processed as described for the other samples. To obtain accurate midpoint of the curves from raw TRF data requires the least square fit of the sigmoideal transition model (Equation 1).
(TIF)

Figure S6 The dissociation and unfolding of rPrP^Sc^ monitored by CDI at different concentrations. The (**a**) row data with TRF or (**b**) values of apparent fractional change (Fapp) at each concentration of Gdn HCl in each dilution are mean ± SEM obtained from triplicate CDI measurements. Note the logaritmic scale in the plot **A** that was necessary due to the 1000-fold range of TRF values but made the manuall estimate of the Gdn HCl_1/2_ difficult. To obtain accurate midpoint of the curves from raw TRF data, we used the least square fit of the sigmoideal transition model (Equation 1) or Fapp transformation. Both methods gave indentical results.
(TIF)

Figure S7 The relationship between duration of the disease and (**a**) concentration of sPrPSc or (**b**) change in the stability of PrP^Sc^ after PK digestion in all sCJD patients (n = 46). The regression analysis was performed by using data from (**a**) **Figure 3** and **(b)** **Figure 6**.
(TIF)
