# Supplementary figures and images for: Protease-Sensitive Conformers in Broad Spectrum of Distinct PrPSc Structures in Sporadic Creutzfeldt-Jakob Disease Are Indicator of Progression Rate
Source: PLoS Pathog. 2011 Sep 8;7(9):e1002242. doi: 10.1371/journal.ppat.1002242 (PMC3169556; doi:10.1371/journal.ppat.1002242)

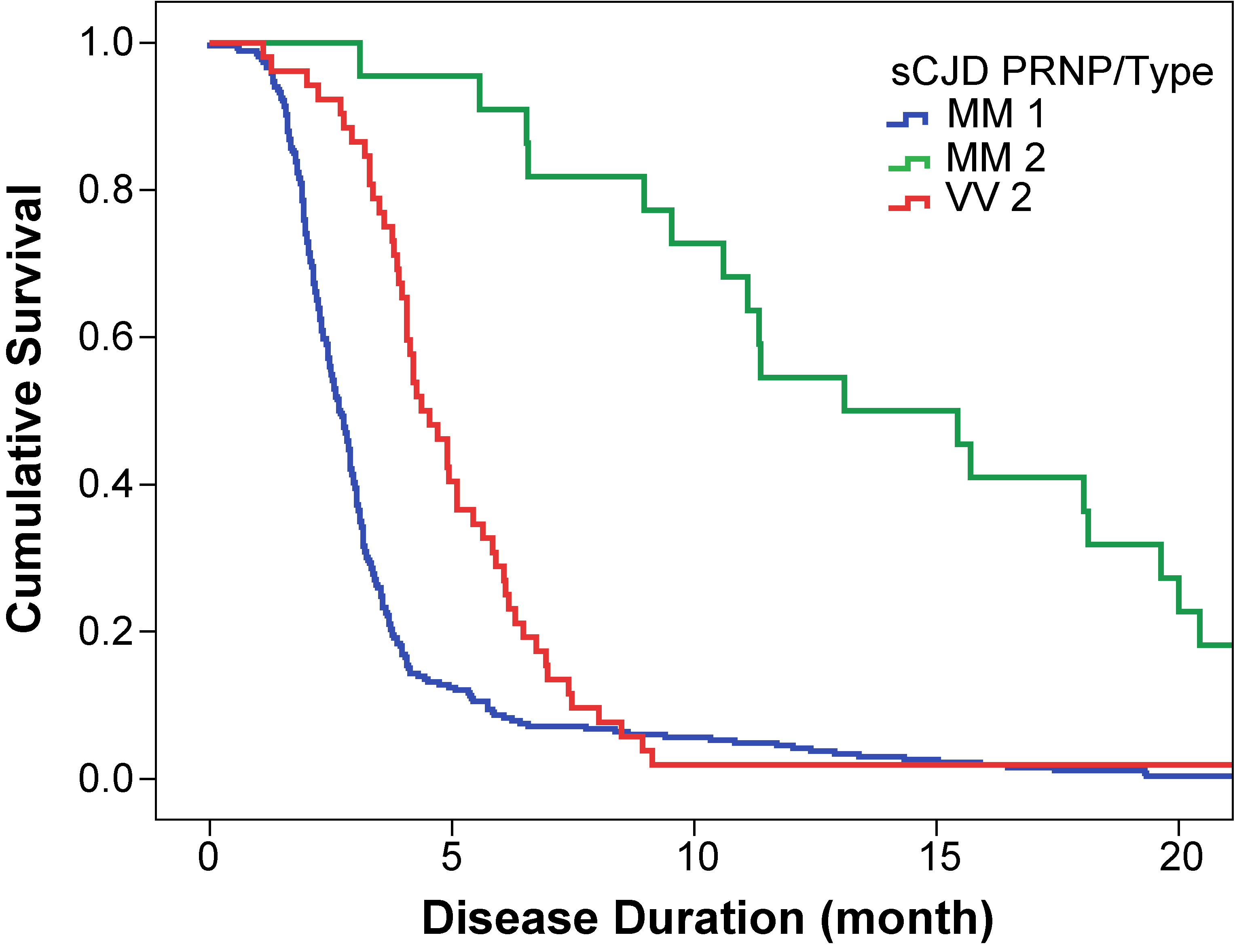

Supplement: Figure S1 — Kaplan-Meier cumulative survival analysis of 340 sCJD cases homozygous for either methionine (n = 288) or valine (n = 52) in codon 129 of PRNP gene from which were selected the 46 cases described in this paper. The sCJD cases carrying pure type 1 PrPSc(129 M) (n = 266) have significantly shorter disease duration than those with type 2 PrPSc(129 M) (n = 22, P<0.001). The intermediate duration of the disease observed in sCJD cases with type 2 PrPSc(129 V) (n = 52) is significant compared with type 1 PrPSc(129 M) (P<0.001) or type 2 PrPSc(129 M) (P<0.001). (TIF) [file ppat.1002242.s001.tif]

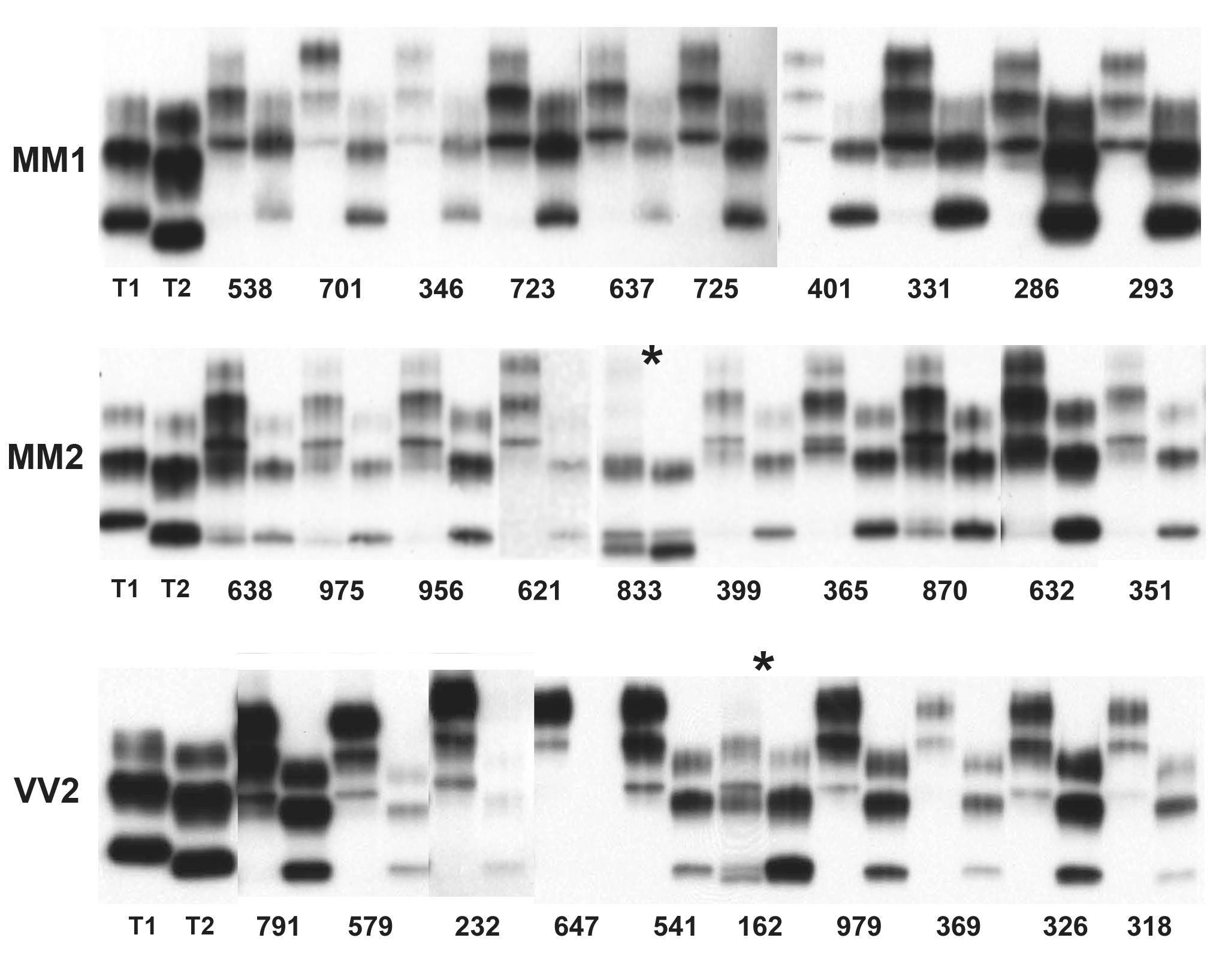

Supplement: Figure S2 — Typical WB analysis of PrPSc and rPrPSc in sCJD cases. PrPSc from 5% brain homogenate in PBS, pH 7.4, containing 1% Sarcosyl was precipitated with PTA either before (left lanes) or after (right lanes) digestion with 50 µg/ml of PK at 37°C for 1 h. Note the 19 and 17 kD doublets of unglycosylated bands of PrPSc in MM2 Case #7-927 and VV2 Case #8-848. The rPrPSc bands in Case VV2 9-434 became visible only after prolonged exposure (data not shown). Internal controls of type 1 (T1) or type 2 (T2) rPrPSc(129 M) were incorporated in each WB. (TIF) [file ppat.1002242.s002.tif]

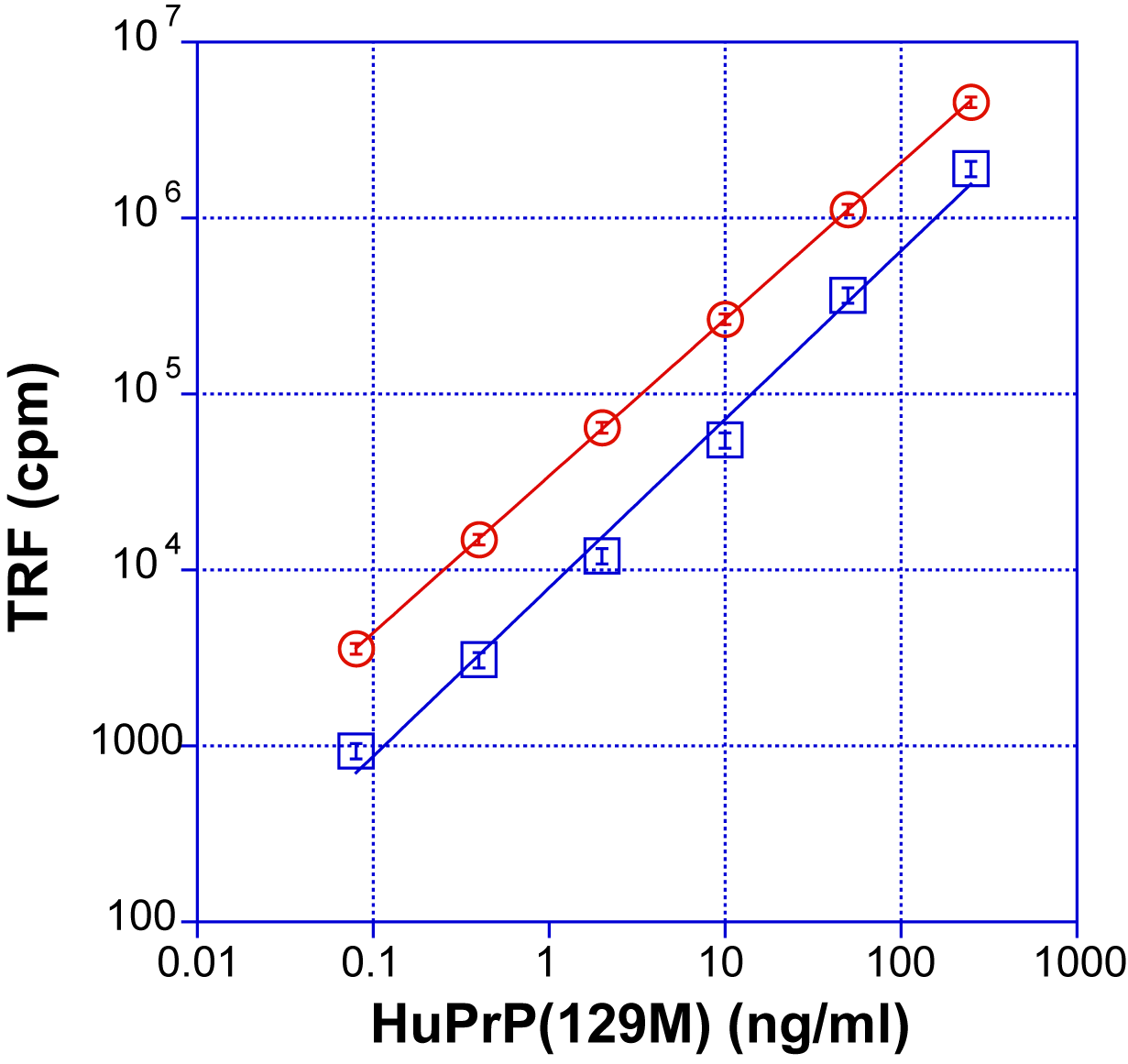

Supplement: Figure S3 — Calibration of CDI with (squares) full length (PrP23–231, 129 M) or (circles) truncated (PrP90–231, 129 M) prion protein. The truncated (PrP90–231, 129 M) prion protein corresponds to the human brain PrP 27–30 after proteinase K treatment. Time-resolved fluorescence (TRF) is reported in counts per minute (cpm) from triplicate measurement ± SEM. The initial concentrations of recombinant human PrP(23–231) and PreP(90–231) were calculated from absorbance at 280 nm and molar extinction coefficient 56650 M−1 cm−1 and 21640 M−1 cm−1, respectively. (TIF) [file ppat.1002242.s003.tif]

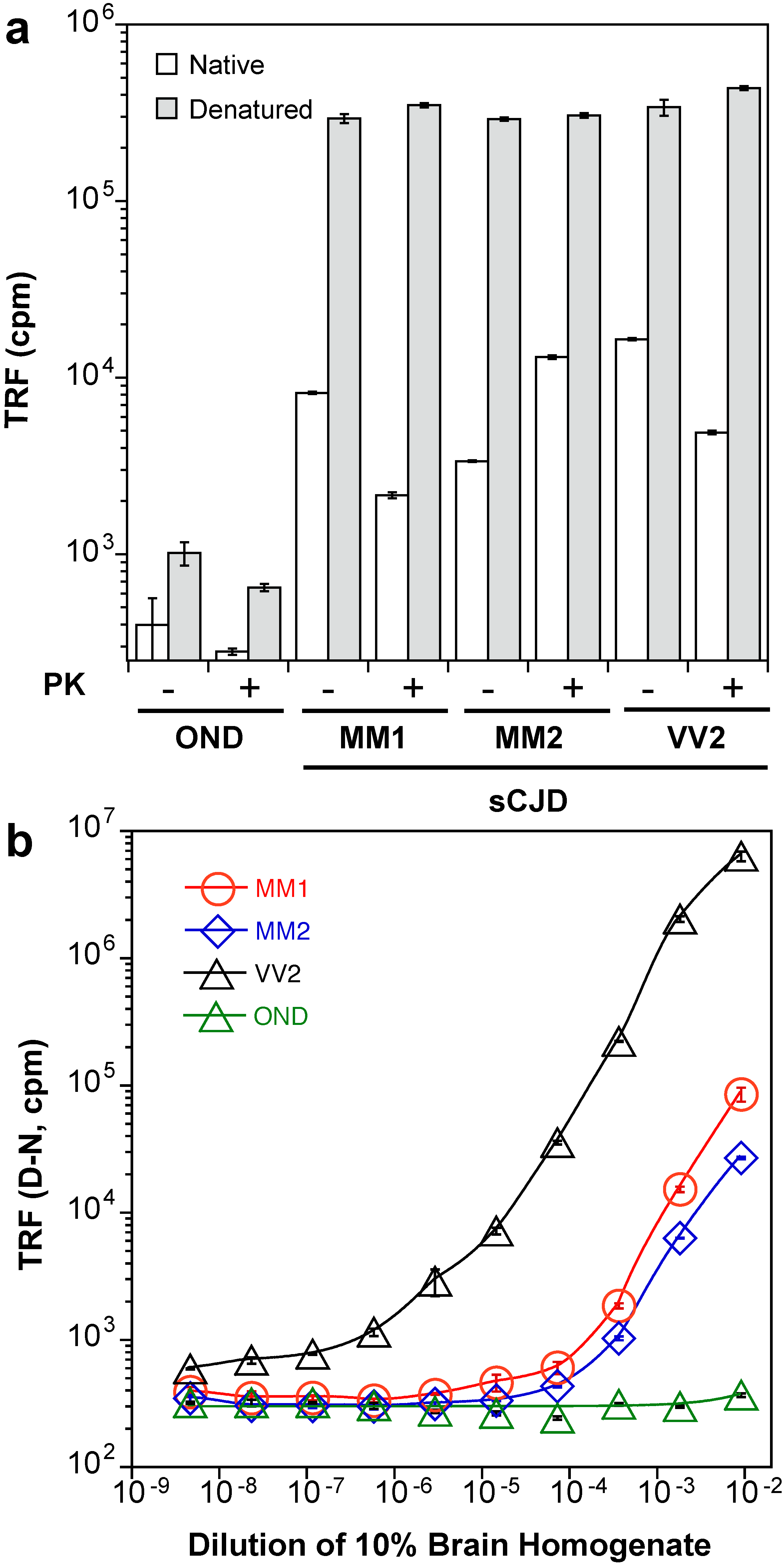

Supplement: Figure S4 — The (a) raw time-resolved fluorescence (TRF) data and (b) end-point sensitivity in detection of sCJD PrPSc with CDI before and after proteinase K treatment in different cases of sCJD and a case of other neurological disorder (OND). To obtain values for total PrPSc, CDI was performed in an aliquot of brain homogenate that was precipitated in the presence of a protease inhibitor cocktail with PTA. To obtain CDI readings for rPrPSc, samples were treated with PK at concentration equivalent to 3 IU/ml (100 µg/ml) of 10% brain homogenate for one hour at 37°C and precipitated with PTA after blocking PK with the protease inhibitor cocktail. The 8H4 mAb was used {Zanusso, 1998 #4838} for capture and Eu-labeled 3F4 mAb for detection under native (N) and denatured (D) conditions {Safar, 2005 #6826;Safar, 2002 #5989;Safar, 1998 #4776}. The (D – N) values of time-resolved fluorescence (TRF) measured in counts per minute (cpm) are directly proportional to the concentration of PrPSc [Safar, 2005 #6826;Safar, 2002 #5989;Safar, 1998 #4776]. Data points and bars represent average ± standard deviation (SD) obtained from three or four independent measurements. (TIF) [file ppat.1002242.s004.tif]

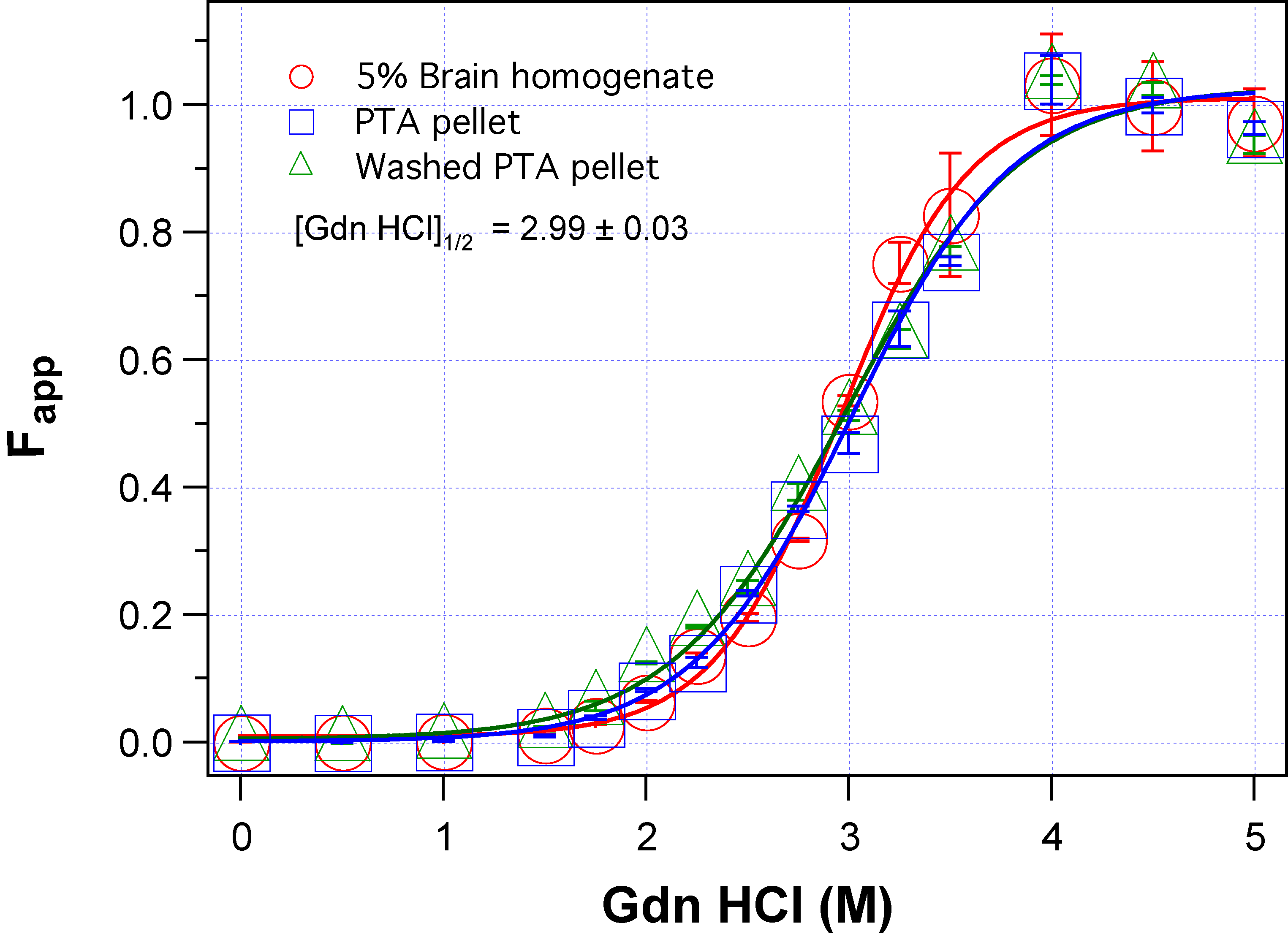

Supplement: Figure S5 — The dissociation and unfolding of PrPSc(129 M) monitored by CDI in 5% brain homogenate (circles), in PTA pellet (squares), and washed PTA pellet (triangles). The brain homogenate and PTA precipitation was performed as described in the Method section. For wash, the PTA pellet was resuspended in 1 ml of H2O containing protease inhibitors, spun at 14,000 G for 30 min, and then processed as described for the other samples. To obtain accurate midpoint of the curves from raw TRF data requires the least square fit of the sigmoideal transition model (Equation 1). (TIF) [file ppat.1002242.s005.tif]

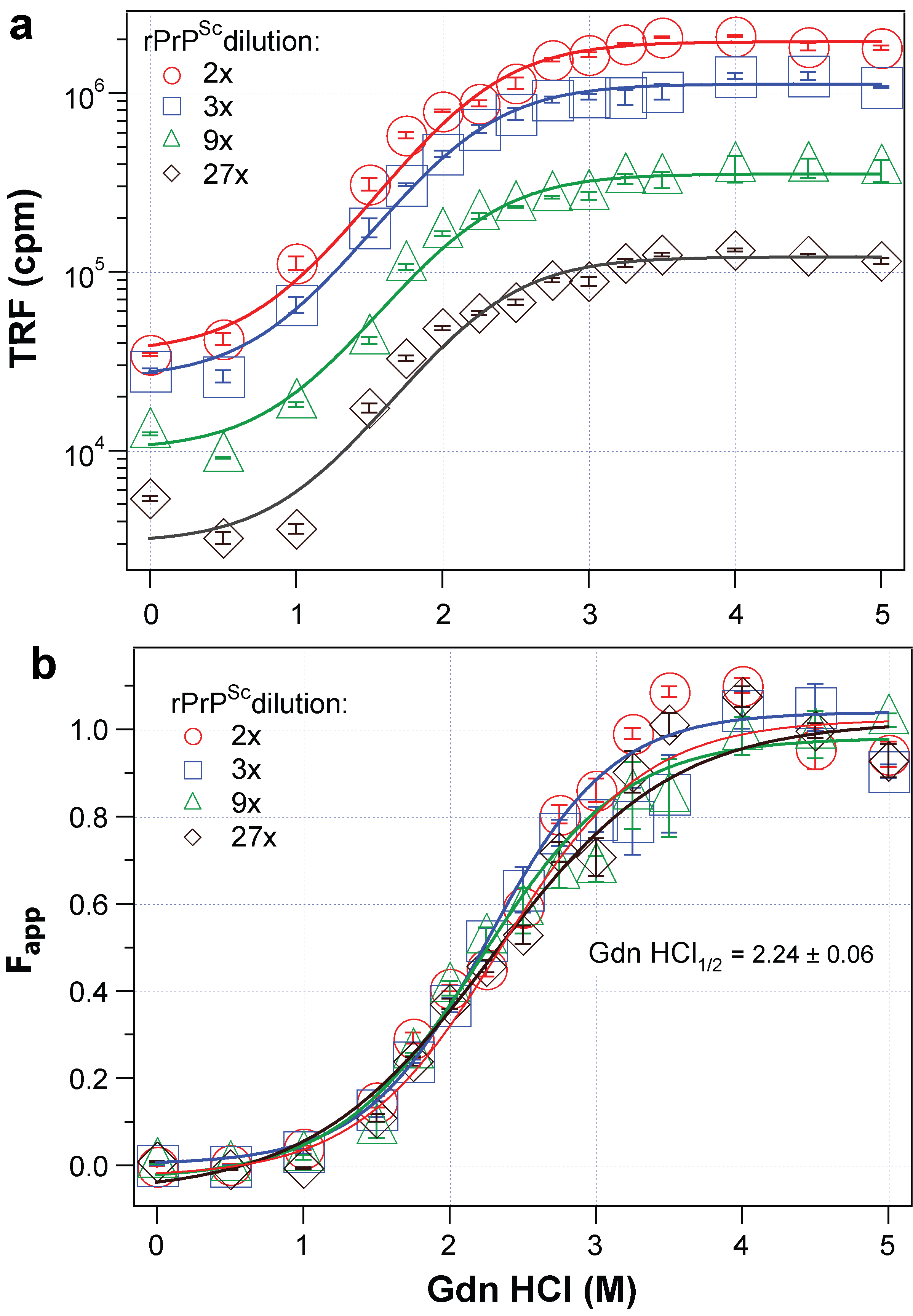

Supplement: Figure S6 — The dissociation and unfolding of rPrPSc monitored by CDI at different concentrations. The (a) row data with TRF or (b) values of apparent fractional change (Fapp) at each concentration of Gdn HCl in each dilution are mean ± SEM obtained from triplicate CDI measurements. Note the logaritmic scale in the plot A that was necessary due to the 1000-fold range of TRF values but made the manuall estimate of the Gdn HCl1/2 difficult. To obtain accurate midpoint of the curves from raw TRF data, we used the least square fit of the sigmoideal transition model (Equation 1) or Fapp transformation. Both methods gave indentical results. (TIF) [file ppat.1002242.s006.tif]

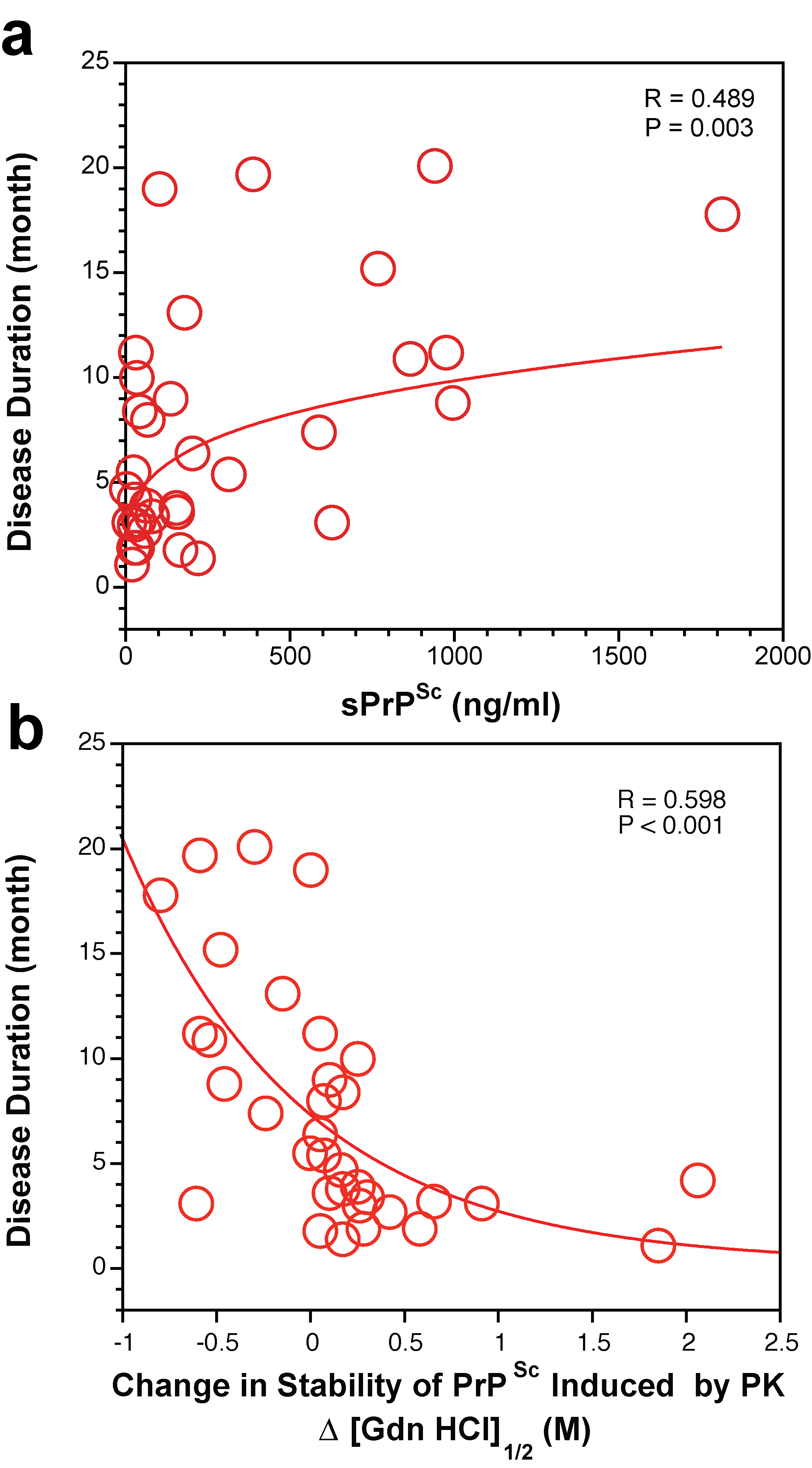

Supplement: Figure S7 — The relationship between duration of the disease and (a) concentration of sPrPSc or (b) change in the stability of PrPSc after PK digestion in all sCJD patients (n = 46). The regression analysis was performed by using data from (a) Figure 3 and (b) Figure 6 . (TIF) [file ppat.1002242.s007.tif]
